# Catatonia in the Context of Emerging Psychosis: Diagnostic and Therapeutic Challenges in Adolescent Psychiatry

**DOI:** 10.7759/cureus.88393

**Published:** 2025-07-20

**Authors:** Alina Kang, Anthony J Maristany, Fayeza Malik, Edmi Y Cortes, Jeremy Hsiang

**Affiliations:** 1 Psychiatry and Behavioral Sciences, University of Miami Miller School of Medicine, Miami, USA; 2 Psychiatry and Behavioral Sciences, University of Miami Miller School of Medicine, Jackson Memorial Hospital, Miami, USA

**Keywords:** adolescent catatonia, cannabis use, child and adolescent psychiatry, lorazepam challenge test, schizophrenia and other psychotic disorders

## Abstract

Catatonia is a complex neuropsychiatric syndrome that can often be difficult to diagnose in adolescents, particularly when it co-occurs with emerging psychosis. Timely diagnosis is crucial, as delayed treatment may lead to serious complications or delay in treatment. We present the case of a 17-year-old Haitian-American male with no prior medical or psychiatric history who was hospitalized for waxing and waning altered mental status. His presentation included mutism, inappropriate smiling, and posturing. Initial workup was unrevealing, but a lorazepam challenge led to transient improvement, supporting a diagnosis of catatonia. He was treated with lorazepam and antipsychotics, with fluctuating response. Over the following eleven months, he developed persistent psychotic symptoms and was ultimately diagnosed with schizophrenia.

Our case illustrates the diagnostic challenges and therapeutic complexities of managing catatonia in the context of emerging psychosis in youth. It highlights the need for high clinical suspicion, multidisciplinary collaboration, and culturally sensitive care. The patient’s history of substance use and brief improvement with naloxone during his initial presentation also raises consideration of substance-related contributions. Importantly, this case underscores the necessity of prompt empiric treatment in diagnostically ambiguous presentations, as early intervention can improve outcomes. Further research is warranted to refine diagnostic strategies and treatment protocols for pediatric catatonia, particularly when occurring in the setting of first-episode psychosis.

## Introduction

A diagnosis of catatonia per the Diagnostic and Statistical Manual for Mental Disorders (DSM-5) requires the presence of three or more of twelve specific psychomotor features, which include mutism, posturing, stupor, echolalia, and waxy flexibility, among others. While historically associated with schizophrenia, catatonia is now recognized across a wide range of psychiatric and medical conditions, including mood disorders, autoimmune encephalitis, substance use, and toxic-metabolic disturbances [1,2]. In pediatric and adolescent populations, catatonia is estimated to have a prevalence of 5.5% in a psychiatry outpatient setting [3]. The clinical picture is often complicated by a significant overlap with other conditions that cause altered mental status; both delirium and especially intoxication are more prevalent conditions in this population and can present with psychomotor slowing, leading to frequent underdiagnosis of catatonia [4]. Cultural factors, including stigma around mental health and reluctance to engage with psychiatric services, may further delay and complicate the diagnostic process.

Timely identification is critical, as catatonia can rapidly progress to life-threatening complications such as malignant catatonia, which includes autonomic instability. A key diagnostic and therapeutic tool is the “benzodiazepine challenge”, where a benzodiazepine (typically lorazepam 1-2 mg) leads to rapid and dramatic improvement in catatonic symptoms. In adolescents, catatonia may also signal the early stages of a primary psychotic disorder such as schizophrenia or schizoaffective disorder [5].

We present a diagnostically challenging case of a 17-year-old male who developed waxing and waning altered mental status, ultimately diagnosed as first-episode psychosis with catatonia. This case report aims to address a gap in the literature by detailing a complex presentation and emphasizing the need for early recognition, multidisciplinary collaboration, and culturally informed care in the evaluation of acute psychiatric symptoms in youth.

## Case presentation

This is a 17-year-old Haitian-American male with no prior psychiatric or medical history who was brought to the emergency department by his mother for waxing and waning altered mental status (AMS) over the preceding seven days. According to the family, he exhibited episodes of unresponsiveness, minimal verbal output, inappropriate smiling, and prolonged staring spells. Over the past several months, he had become increasingly socially withdrawn, particularly following a physical altercation with friends that resulted in dental trauma. One week prior to presentation, his sister noted that he had begun talking to himself with further decline in social engagement.

**Figure 1:**
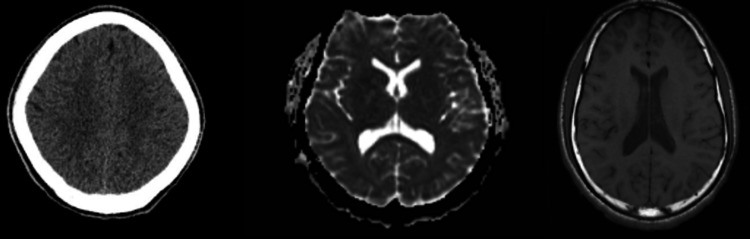
(Left) Non-contrast CT brain showing no evidence of acute intracranial hemorrhage, mass effect, midline shift, or hydrocephalus. (Center) MRI brain without contrast demonstrating normal parenchymal signal intensity on T1- and T2-weighted sequences, with no signs of acute infarct, mass lesion, or demyelinating process. (Right) MRI brain with contrast showing no abnormal enhancement.

Upon initial evaluation in the emergency department, the patient was awake but minimally responsive, with a Glasgow Coma Scale (GCS) of 8. He was nonverbal, displayed inappropriate smiling, and had pinpoint pupils. Initial laboratory evaluation, including basic metabolic panel, liver function tests, complete blood count, urinalysis, and urine toxicology, was unremarkable (Tables 1-4). A non-contrast computed tomography (CT) of the brain showed no acute intracranial abnormalities (Figure 1). Notably, after administration of 2 mg of intravenous naloxone, the patient briefly became more interactive and admitted to recent oxycodone use, raising concern for potential opioid intoxication. He was admitted to the pediatric intensive care unit (PICU) for tachycardia, tachypnea, and respiratory monitoring for risk of apnea.

**Table 1:** Complete Metabolic Panel eGFR: estimated glomerular filtration rate; FAS: full age spectrum; EKFC: European Kidney Function Consortium

Lab Values	Normal Range	Patient Values
Glucose	74 - 106 mg/dL	93 mg/dL
Sodium	137 - 145 mmol/L	140
Potassium	3.6 - 5.0 mmol/L	3.8 mmol/L
Chloride	98 - 107 mmol/L	106 mmol/L
CO_2_	22 - 30 mmol/L	28 mmol/L
Anion gap	7 - 15	6
Osmolality calculated	275 - 295 mOsm/kg	280 mOsm/kg
BUN level	9 - 20 mg/dL	13 mg/dL
Creatinine level	0.66 - 1.25 mg/dL	0.70 mg/dL
Calcium level	8.4 - 10.2 mg/dL	9.9 mg/dL
Total protein	6.3 - 8.2 g/dL	8.1 g/dL
Albumin level	3.5 - 5.0 g/dL	4.6 g/dL
Total bilirubin	0.2 - 1.3 mg/dL	0.8 mg/dL
AST (SGOT)	17 - 59 unit/L	23 unit/L
ALT (SGPT)	9 - 45 unit/L	13 unit/L
Alkaline phosphatase	38 - 126 unit/L	125 unit/L
Magnesium level	1.7 - 2.2 mg/dL	2.2 mg/dL
Phosphorous	2.5 - 4.5 mg/dL	2.9 mg/dL
eGFR FAS-EKFC	> 90	> 90
Acetone level	None detected	None detected
CPK	56 - 433 unit/L	141 unit/L

**Table 2:** Complete Blood Count MCV: mean corpuscular volume; MCH: mean corpuscular hemoglobin; MCHC: mean corpuscular hemoglobin concentration; RDW-CV: red cell distribution width-coefficient of variation; MPV: mean platelet volume; NRBC: absolute nucleated red blood cell count

Lab Values	Normal Range	Patient Values
WBC count	4.0 - 10.5 × 10^3^/mcL	4.3 × 10^3^/mcL
RBC count (×10^6^)	3.80 - 4.90 × 10^6^/mcL	4.60 × 10^6^/mcL
Hemoglobin	11.1 - 14.6 g/dL	13.6 g/dL
Hematocrit	33.2 - 43.4%	41.9%
MCV	79.9 - 95.0 fL	91.1 fL
MCH	26.5 - 31.7 pg	29.6 pg
MCHC	32.0 - 35.2 g/dL	32.5 g/dL
RDW-CV	11.0 - 15.0%	12.0%
Platelet	140 - 400 × 10^3^/mcL	228 × 10^3^/mcL
MPV	9.4 - 16.4 fL	9.8 fL
NRBC%	0.0 - 0.0%	0.0%
NRBC (Abs)	0.00 - 0.00/100 WBC	0.00/100 WBC
Neutrophil (%)	36.0 - 70.0%	50.5%
Lymphocyte (%)	16.0 - 43.0%	37.6%
Monocyte (%)	6.0 - 12.0%	9.2%
Eosinophil (%)	0.0 - 5.0%	1.8%
Basophil (%)	0.0 - 1.2%	0.7%
Immature granulocyte (%)	0.0 - 1.0%	0.2%
Absolute neutrophil	2.0 - 6.0 × 10^3^/mcL	2.2 × 10^3^/mcL
Absolute lymphocyte	1.1 - 2.7 × 10^3^/mcL	1.6 × 10^3^/mcL
Absolute monocyte	0.3 - 0.9 × 10^3^/mcL	0.4 × 10^3^/mcL
Absolute eosinophil	0.10 - 0.50 × 10^3^/mcL	0.08 × 10^3^/mcL
Absolute basophil	0.01 - 0.20 × 10^3^/mcL	0.03 × 10^3^/mcL
Absolute immature granulocyte	0.00 - 0.10 × 10^3^/mcL	0.01 × 10^3^/mcL

**Table 3:** Urinalysis UA: urinalysis

Lab Values	Normal Range	Patient Values
Urine color	Yellow	Yellow
UA clarity	Clear	Clear
Urine specific gravity	1.001 - 1.035	1.025
Urine pH	4.6 - 8.0	8.0
Urine protein	Negative	Negative
Urine glucose	Negative	Negative
Urine ketones	Negative	Negative
Urine bilirubin	Negative	Negative
Urine blood	Negative	Negative
Urine urobilinogen	Negative	Negative
Urine nitrite	Negative	Negative
Urine leukocyte esterase	Negative	25 units/mcL
Urine WBC	0 - 5/HPF	4/HPF
Urine RBC	0 - 4/HPF	1/HPF
Urine bacteria	0/HPF	0/HPF
Urine squamous epithelial cells	0 - 5/HPF	2/HPF

**Table 4:** Urine Toxicology

Lab Values	Normal Range	Patient Values
Acetaminophen level	10.0 - 30.0 mg/L	< 10.0 mg/L
Ethanol level	0 - 10 mg/L	< 10 mg/L
Cocaine & metabolites	Not detected	Not detected
Cannabinoid	Not detected	Not detected
Benzodiazepine class	Not detected	Not detected
Opiate class	Not detected	Not detected
Amphetamine class	Not detected	Not detected
Barbiturate class	Not detected	Not detected
Methadone	Not detected	Not detected
Salicylate level	0.0 - 25.0 mg/L	< 1.0 mg/L
Urine fentanyl	Not detected	Not detected
Methanol	None detected	None detected
Isopropanol	None detected	None detected
Oxycodone	Not detected	Not detected

During his PICU stay, his mental status continued to fluctuate. Neurology was consulted, and an electroencephalogram (EEG) revealed generalized slowing consistent with encephalopathy. Magnetic resonance imaging (MRI) of the brain (Figure 1) and cerebrospinal fluid (CSF) studies, including autoimmune and infectious panels, were unremarkable (Tables 5-7). Psychiatry was consulted to evaluate for catatonia and psychosis. On exam, the patient met criteria for catatonia as per the DSM-5, with an initial Bush Francis Catatonia Rating Scale (BFCRS) score of 17. A lorazepam challenge resulted in rapid, temporary clinical improvement, with a reduction in BFCRS score to 8.

**Table 5:** CSF Fluid Analysis

Lab Values	Normal Range	Patient Values
CSF appearance	Clear	Clear
CSF supernatant	Clear	Clear
CSF WBC	0 - 5 mm^3^	1 mm^3^
CSF RBC	0 - 0 mm^3^	0 mm^3^
CSF glucose	40 - 70 mg/dL	56 mg/dL
CSF total protein	5 - 40 mg/dL	< 10 mg/dL
IgG CSF	0.00 - 3.39 mg/dL	1.20 mg/dL
CSF neutrophils Seg %	0 - 6%	0%
CSF lymphocytes %	40 - 80%	78%
CSF monocytes %	15 - 45%	22%
CSF diff type	Manual	Manual
IgG serum	650.00 - 1,600.00 mg/dL	1,311.80 mg/dL

**Table 6:** Infectious Panel VDRL-CSF: venereal disease research laboratory test of cerebrospinal fluid

Lab Values	Normal Range	Patient Values
*E. coli* K1	Not detected	Not detected
Haemophilus influenzae	Not detected	Not detected
Listeria monocytogenes	Not detected	Not detected
Neisseria meningitidis	Not detected	Not detected
Streptococcus agalactiae	Not detected	Not detected
Streptococcus pneumoniae	Not detected	Not detected
Cytomegalovirus (CMV)	Not detected	Not detected
Enterovirus	Not detected	Not detected
Human herpes virus 6 (HHV-6)	Not detected	Not detected
Herpes simplex virus 1 (HSV-1)	Not detected	Not detected
Herpes simplex virus 2 (HSV-2)	Not detected	Not detected
Human parechovirus	Not detected	Not detected
Varicella zoster virus (VZV)	Not detected	Not detected
*Cryptococcus neoformans*/*Cryptococcus gattii*	Not detected	Not detected
VDRL-CSF	Non-reactive	Non-reactive

**Table 7:** Autoimmune Panel SSA: anti-Ro; SSB: anti-La

Lab Values	Normal Range	Patient Values
ANA speckled	Negative	Present
ANA nucleolar	Negative	Present
ANA speck titer	< 1:80	1:160
ANA nucleolar titer	< 1:80	1:80
Anti-SSA	Negative	Negative
Anti-SSB	Negative	Negative
Anti-Smith	Negative	Negative
Anti-RNP	Negative	Negative
Anti-SMRNP antibody	Negative	Negative
Anti-ribosomal P antibody	Negative	Negative
Anti-Scleroderma 70	Negative	Negative
Anti-JO 1 antibody	Negative	Negative
Anti-chromatin antibody	Negative	Negative
Anti-centromere antibody	Negative	Negative
Anti-Ds DNA	0 - 4 IU/mL	< 1 IU/mL
Anti-Ds DNA interpretation	Negative	Negative
Serum albumin	3,500 - 5,200 mg/dL	3,946 mg/dL
CSF albumin	0.00 - 34.99 mg/dL	6.20 mg/dL
CSF ratio	0.070 - 0.270	0.194
CSF index	0.000 - 0.770	0.584
IgG	650.00 - 1,600 mg/dL	1,088.90 mg/dL
IgA	68.00 - 379.00 mg/dL	196.20 mg/dL
IgM	41.00 - 251.00 mg/dL	59.20 mg/dL

The patient was initiated on scheduled intravenous lorazepam and later transitioned to oral dosing. Risperidone was started due to concern for worsening psychotic features. Over several days, the patient demonstrated gradual improvement in responsiveness, interaction, and orientation. However, he continued to exhibit inappropriate affect, disorganized thought processes, and delusional statements (e.g., stating he was a vampire). His catatonic features waxed and waned, with regression noted after missed doses of lorazepam. Ultimately, the patient was diagnosed with first-episode psychosis with catatonia, likely within the spectrum of schizoaffective disorder or schizophrenia. His lorazepam was tapered off, and he was discharged on oral olanzapine with close outpatient psychiatric follow-up.

The patient had no documented psychiatric or medical history, nor prior hospitalizations. Family psychiatric history was noncontributory, though his sister noted that mental health was seldom discussed in the household, raising the possibility of unrecognized or unreported conditions. Although his mother denied concerns for substance use and his initial urine toxicology screen was negative for commonly screened substances, the patient reported that substances - specifically oxycodone, alcohol, and cannabis - were a significant problem in his life.

During follow-up visits in the first two to three months post-discharge, the patient exhibited selective mutism and inappropriate smiling. He appeared psychotic with internal preoccupations and a lack of eye contact. His mother reported his lack of communication with family members and poor sleep. The patient was subsequently started on trazodone for sleep and switched from olanzapine to risperidone, due to his lack of improvement in psychosis.

Over the following three months, the patient’s psychotic symptoms persisted despite a gradual uptitration of risperidone. As a result, risperidone was discontinued, and haloperidol was initiated. Despite dose escalation over the following four months, there continued to be minimal clinical improvement; the patient continued to exhibit active psychosis, including responding to internal stimuli, inappropriate smiling, and disorganized speech. Approximately nine months after discharge from the inpatient unit, he was diagnosed with schizophrenia. The patient was recommended to transition to clozapine due to treatment resistance; however, the patient and his mother declined, expressing concerns about the regular blood monitoring requirement. The full hospital course, events, and interventions are detailed in Table 8.

**Table 8:** Hospital Course, Events, and Interventions ED: emergency department; AMS: altered mental status; PICU: pediatric intensive care unit; LP: lumbar puncture; PO BID: by mouth or orally twice a day; GCS: Glasgow Coma Scale; EEG: electroencephalogram; CAAP: Colposcopy, Atypical Cells of Undetermined Significance, and Pap smear; BFCRS: Bush Francis Catatonia Rating Scale; UTox: urine toxicology; CXR: chest X-ray; PO BID: per os twice a day

Hospital Day	Key Events/Findings	Interventions/Treatments
Day 0	Presented to ED with a 7-day history of waxing/waning AMS, pinpoint pupils, and smiling inappropriately. GCS 8.	Administered naloxone; admitted to PICU for monitoring. Initial labs, CT, and CXR were unremarkable. Urine tox screen negative.
Day 1	Minimally responsive. EEG showed generalized slowing. Neurology and Psychiatry were consulted. Catatonia suspected.	Planned for MRI and LP. Delirium precautions were initiated. No seizures on EEG. No focal neurologic findings.
Day 2	Increased activity, inappropriate speech, flight of ideas. MRI obtained (normal). Psychiatry favored catatonia/psychosis.	Initiated IV lorazepam 2 mg TID. Ordered 1:1 sitter. Continued neuro checks. Started risperidone 0.5 mg PO BID.
Day 3	Improved interactivity. Stated nonsensical answers. Occasional catatonic features observed (posturing, mutism).	Maintained lorazepam 2 mg TID and risperidone 0.5 mg BID. Continued neuro/psych follow-up. PO intake improved.
Day 4	Sustained improvement per family (~85% baseline). Alert, but odd statements. Catatonia and psychosis remained likely.	Continued lorazepam 2 mg TID and risperidone 0.5 mg BID. Encouraged a regular diet. PO hydration was adequate.
Day 5	Refused PO lorazepam, regressed in responsiveness. Seen sleeping, drooling, and unresponsive to questions.	Maintained lorazepam 2 mg TID and risperidone 0.5 mg BID. Educated family.
Day 6	Patient stated he was a “vampire”, refused food due to “no blood”. He was found covering his face and avoiding light.	Increased risperidone to 0.5 mg qAM, 1 mg qHS. Increased lorazepam to 3 mg TID. Continued monitoring.
Day 7 - 9	Thought blocking and disorganized speech. The patient was intermittently compliant with PO food intake and medications.	Continued lorazepam 3 mg TID and risperidone 0.5 mg qAM, 1 mg qHS.
Day 10 (Transfer to CAAP)	The patient was unresponsive to the staff, intermittently laughing inappropriately. After 1 mg of lorazepam administration, the patient was seen sitting up, staring, and smiling.	Continued lorazepam 3 mg TID and risperidone 0.5 mg qAM, 1 mg qHS.
Day 11	Required IM lorazepam due to refusal of oral lorazepam. Refused to answer questions. Low oral intake.	Continued lorazepam 3 mg TID and risperidone 0.5 mg qAM, 1 mg qHS.
Day 12	Intense sideward and fixed gaze. Minimally responsive during assessment. Nonverbal. Smiling inappropriately. Responded to internal stimuli. Rigid arms, waxy flexibility. Refused to eat, drink, or make eye contact. BFCRS: 14	Continued lorazepam 3 mg TID or IM lorazepam 2 mg if PO refusal. Discontinued risperidone and started olanzapine 2.5 mg QHS. Started N-acetylcysteine 600 mg TID.
Day 13	Received IM olanzapine and diphenhydramine for exposing himself and masturbating in front of others. Verbigeration and inappropriate smiling. No rigidity or waxy flexibility. BFCRS: 6	Continued lorazepam 3 mg TID or IM lorazepam 2 mg if PO refusal. Continued olanzapine 2.5 mg QHS. Continued N-acetylcysteine 600 mg TID.
Day 14	Agitated overnight. Superficially cooperative and stated that drugs, specifically oxycodone, cannabis, and alcohol, were a big problem in his life. However, UTox was negative on admission.	Decreased lorazepam to 2 mg PO TID. Switched olanzapine to olanzapine Zydis 10 mg PO daily. Continued N-acetylcysteine 600 mg TID.
Day 15	Sat with bilateral legs raised, staring at the floor. Nonverbal. Preoccupied with delusions related to vampires and werewolves. BFCRS: 10	Decreased lorazepam to 2 mg PO BID. Increased olanzapine Zydis to 15 mg PO daily. Continued N-acetylcysteine 600 mg TID.
Day 16	The patient was interactive and responsive. Responded to internal stimuli with persistent delusions. Found to be eating a muffin wrapper.	Decreased lorazepam to 1 mg PO BID. Increased olanzapine Zydis to 20 mg PO daily. Continued N-acetylcysteine 600 mg TID.
Day 17	Little concern for catatonia, but remained with symptoms of psychosis. Disorganized thought process, smiling inappropriately, persistent delusion.	Continued lorazepam 1 mg PO BID. Continued olanzapine Zydis 20 mg PO daily. Continued N-acetylcysteine 600 mg TID.
Day 18	Catatonia was mostly resolved. Less disorganized thought process. Engaged briefly in conversations. Remained sexually preoccupied but was easily redirectable.	Final dose of lorazepam 1 mg PO administered. Continued olanzapine Zydis 20 mg PO daily.
Day 19 (Discharge from CAAP)	Catatonia symptoms resolved. Endorsed improvement in mood symptoms. Deemed psychiatrically stable for discharge.	Discharged with diagnoses of unspecified psychosis and resolved catatonic disorder due to a physiological condition. Discharged on olanzapine Zydis 20 mg PO daily. Instructed mother to purchase N-acetylcysteine 600 mg PO TID over the counter.
Outpatient follow-ups (~1 and 3 months post-discharge)	Selective mutism and inappropriate smiling. Appeared psychotic with internal preoccupations and a lack of eye contact. Mother reported a lack of communication with family and poor sleep.	Discontinued olanzapine Zydis. Started risperidone 1 mg PO BID. Started trazodone 50 mg PO QHS.
Outpatient follow-up (~4 months post-discharge)	Interactive and verbal, but was inappropriately smiling and responding to internal stimuli. Poor eye contact. Reported feeling “sad”. Mother reported increased communication with family members.	Increased risperidone to 2 mg PO BID. Continued trazodone 50 mg PO QHS.
Outpatient follow-up (~5.5 months post-discharge)	Internally preoccupied and not engaged with the provider. Tangential thought process. Smiling inappropriately. Patient without medications for 2 weeks. Mother reported the patient is “not doing well”.	Increased risperidone to 3 mg PO BID. Started benztropine 1 mg PO BID. Continued trazodone 50 mg PO QHS. Mother was educated on the importance of a strict medication administration schedule.
Outpatient follow-up (~6.5 months post-discharge)	Internally preoccupied and smiling inappropriately. Poor eye contact. Briefly interacted to state he wanted to work for Amazon.	Discontinued risperidone 3 mg PO BID. Started haloperidol 5 mg PO BID. Continued benztropine 1 mg PO BID. Continued trazodone 50 mg PO QHS.
Outpatient follow-up (~7.5 months post-discharge)	Internally preoccupied and smiling inappropriately. Made nonsensical statements with loose associations. Per the mother, the patient acted aggressively and talked to himself at home.	Suggested clozapine if no improvement with haloperidol, which the mother declined. Increased haloperidol to 10 mg PO BID. Increased benztropine to 2 mg PO BID. Continued trazodone 50 mg PO QHS.
Outpatient follow-up (~9 months post-discharge)	The patient ran out of medications due to a missed appointment two weeks ago. Mother provided conflicting information, stating the patient became aggressive and physically hit her, but then later denied this. The patient had internal preoccupations and little communication with family.	Suggested IM haloperidol decanoate, which the mother declined. Increased haloperidol to 15 mg PO BID. Continued benztropine to 2 mg PO BID. Continued trazodone 50 mg PO QHS. ID.
Outpatient follow-up (~10 months post-discharge)	The patient was more interactive with his mother and provider. Voluntary hand spasticity, but no evidence of catatonia. Admitted to hearing voices but was unable to elaborate. Poor eye contact and inappropriate smiling.	Increased haloperidol to 20 mg PO BID. Continued benztropine to 2 mg PO BID. Continued trazodone 50 mg PO QHS. ID.
Outpatient follow-up (~11 months post-discharge)	Remained selectively mute, interacting only with his mother. Internally preoccupied but smiled less than usual. Poor eye contact. Per mother, patient no longer talked to self.	Diagnosed with schizophrenia. Increased haloperidol to 30 mg PO BID. Continued benztropine to 2 mg PO BID. Continued trazodone 50 mg PO QHS. ID.

## Discussion

Catatonia is a complex neuropsychiatric syndrome characterized by a range of motor, behavioral, and autonomic symptoms including mutism, posturing, waxy flexibility, echolalia, echopraxia, and stupor. Although more frequently described in adults, particularly in the context of mood disorders or schizophrenia, catatonia is increasingly recognized in pediatric and adolescent populations. Its presentation in this age group is often subtle, variable, and prone to misdiagnosis, particularly when psychiatric history is absent and the presentation overlaps with medical or toxicologic etiologies [1,4].

This case illustrates a diagnostic challenge involving a 17-year-old male with no prior psychiatric history who presented with waxing and waning AMS, initially raising concerns for a broad list of differential diagnoses encompassing toxic-metabolic encephalopathy, substance-induced delirium, autoimmune encephalitis, and primary psychiatric illness. Additionally, EEG results showed generalized slowing consistent with a presentation of encephalopathy. However, normal subsequent neuroimaging and unremarkable CSF studies rendered a primarily neurologic etiology less likely. Although serial toxicology screens were negative, a brief improvement in alertness following naloxone administration, along with the reported frequent use of oxycodone, raised the possibility of opioid involvement. Current literature shows documented catatonia following withdrawal from psychoactive substances, including opioids [6]. The pathophysiology is thought to involve abrupt changes in neurotransmission, especially after chronic exposure to agents that modulate these systems. Further, opioids have been known to suppress the activity of both excitatory (glutaminergic) and inhibitory (GABAergic) neurotransmission, while catatonia has been hypothesized to be caused by an imbalance of excessive glutamate activity and decreased GABA activity. Therefore, the use of opioids may lead to hypoactivity and contribute to the development of catatonia [7].

The patient’s initial unresponsiveness with intermittent and inappropriate smiling, withdrawal to pain, and resistance to examination raised concern for catatonia. A lorazepam challenge, the standard bedside diagnostic tool, led to transient improvement, supporting the diagnosis [2]. Given this response, early recognition and intervention in adolescent catatonia are critical, as timely treatment may significantly influence long-term outcomes. Notably, catatonia in youth can co-occur with, or serve as a prodrome to, emerging primary psychiatric conditions, including psychotic and mood disorders [8,9].

The case also underscores the importance of cultural context. The patient came from a Haitian-Creole-speaking household with minimal discussion of mental illness. Cultural stigma around psychiatric diagnoses and limited prior engagement with mental health services may have contributed to delays in recognition and treatment. Such factors are critical to consider, especially when communicating diagnoses and involving families in treatment planning [10].

Finally, the patient’s fluctuating responsiveness, regression after missed doses, and ultimate stabilization on lorazepam and risperidone are consistent with catatonia in the setting of a first-episode psychotic disorder. The patient in our case exhibited treatment resistance to multiple antipsychotics, and clozapine was eventually recommended, given its known efficacy in treatment-resistant schizophrenia. Clozapine was declined, and the patient remained on haloperidol, with minimal improvement in his psychotic and catatonic symptoms. The selection of pharmacological treatment for patients with both catatonia and psychotic disorders is crucial, as some antipsychotics used in the management of psychosis may exacerbate catatonic symptoms. Specifically, the use of typical antipsychotics was more frequently associated with adverse effects, emphasizing the need for caution in their use, while clozapine was shown to have beneficial effects on catatonic features [11]. As such, clozapine may be even more efficacious than generally believed for treatment-resistant psychosis with concomitant catatonia. Delays in initiation or patient hesitancy toward starting clozapine often result in prolonged illness and functional decline.

Notably, literature suggests that adolescents presenting with catatonia and psychotic symptoms are at higher risk for developing chronic psychotic conditions if not treated promptly [12]. This case reinforces the importance of early recognition and empiric treatment trials in suspected pediatric catatonia, even in diagnostically ambiguous cases.

## Conclusions

This case highlights the diagnostic challenges of adolescent catatonia, especially in the absence of prior psychiatric history. Early identification and empiric treatment with lorazepam can serve both diagnostic and therapeutic roles. The emergence of psychotic features supported a diagnosis of first-episode psychosis with catatonia. Further research is crucial in order to establish effective and standardized methods for early recognition and treatment. Future studies may explore the relationship between catatonia and first-episode psychosis and reveal the factors associated with positive treatment outcomes.
